# Association of antibiotic exposure with survival in patients with extensive‐stage small cell lung cancer receiving immune checkpoint inhibitor therapy

**DOI:** 10.1111/1759-7714.15172

**Published:** 2023-11-27

**Authors:** Jiaqi Zhong, Dali Xiong, Yu Liu, Shuanghu Yuan

**Affiliations:** ^1^ Clinical Medical College Southwest Medical University Luzhou China; ^2^ Department of Radiation Oncology Shandong Cancer Hospital and Institute, Shandong First Medical University and Shandong Academy of Medical Sciences Jinan China; ^3^ Shandong Provincial Hospital Affiliated to Shandong First Medical University Digestive Endoscopy Center Jinan China; ^4^ Department of Radiation Oncology The Affiliated Cancer Hospital of Zhengzhou University Zhengzhou China

**Keywords:** antibiotic, extensive‐stage small cell lung cancer, gut microbiome, immune checkpoint inhibitor

## Abstract

**Background:**

Immune checkpoint inhibitors (ICIs) have dramatically shifted the therapeutic paradigm of extensive‐stage small cell lung cancer (ES‐SCLC). Antibiotic (ATB) exposure before or during ICI therapy can harm the integrity of the gut microbiome and lead to intestinal dysbiosis, which has a profoundly negative impact on the treatment response for various malignancies. Whether this is applicable to ES‐SCLC remains unclear.

**Methods:**

We retrospectively reviewed the electronic medical records of all patients diagnosed with ES‐SCLC who were treated with ICI‐based immunotherapies from July 2019 to December 2020 at Shandong Cancer Hospital and Institute, China. Outcomes with the use of ATBs before or after the first infusion of ICI, including progression‐free survival (PFS) and overall survival (OS), were investigated using the Kaplan–Meier method. Multivariate analyses were also conducted using a Cox proportional hazards model.

**Results:**

A total of 214 patients were included, among whom 41 (19.2%) received ATBs within 2 months before or after the first initiation of ICI therapy and were assigned to the ATB group. The ATB group showed a shorter median PFS (4.3 vs. 6.3 months; HR = 1.43, 95% CI: 0.97–2.11; *p =* 0.043) and a significantly shorter median OS (6.9 vs. 13 months; HR = 1.47, 95% CI: 0.98–2.20; *p =* 0.033) than the non‐ATB group. In the multivariate analysis, ATB exposure was markedly associated with worse PFS (HR = 1.47, 95% CI: 1.03–2.09, *p =* 0.035) and OS (HR = 1.46, 95% CI: 1.01–2.11, *p =* 0.043).

**Conclusions:**

Our results demonstrate that ATB exposure was significantly associated with worse survival in ES‐SCLC patients who received ICI therapy.

## Graphical abstract

This is the first study to demonstrate the potential harmful effects of ATB exposure in Chinese ES‐SCLC patients receiving anti‐PD1/PD‐L1‐based immunotherapy. Antibiotic exposure leads to worse efficacy of ICIs in ES‐SCLC.

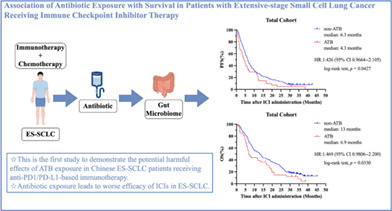

## INTRODUCTION

Small cell lung cancer (SCLC) is an aggressive form of lung cancer characterized by high malignancy and an extremely dismal prognosis.^1^ Extensive‐stage SCLC (ES‐SCLC) accounts for 70% of all SCLCs, defined as tumors with distant metastasis or exceeding the area that can be treated within a radiation field. Patients have a 5‐year survival of only 3%.^2^ The U.S. Food and Drug Administration (FDA) has approved the antiprogrammed cell death‐ligand 1 (PD‐L1) antibody atezolizumab in combination with carboplatin and etoposide as the first‐line treatment for adult patients with ES‐SCLC.^3^ Immune checkpoint inhibitors (ICIs) have shifted the therapeutic paradigm of SCLC, yielding longer median progression‐free survival (mPFS) (5.2 m vs. 4.3 m) and median overall survival (mOS) (12.3 m vs. 10.3 m) than placebo plus carboplatin and etoposide.

A substantial proportion of patients still show no response to ICIs, which suggests that it is of paramount importance to identify reliable predictors which will enable more precise delivery of immunotherapy. Biomarkers such as intertumoral PD‐L1 expression, tumor mutation burden (TMB), tumor‐infiltrating T lymphocytes (TILs) and neoantigens have been identified to predict the efficacy of ICIs,^4^, ^5^, ^6^ but the clinical predictive values are not entirely consistent. Notably, several studies in preclinical models have highlighted the crucial impact of the gut microbiome (GM) in modifying tumor responses to immunotherapy: greater GM diversity and certain bacterial species are linked to improved ICI outcomes,^7^, ^8^, ^9^ showing that the GM plays a key role in regulating the host innate and acquired immune response. Exposure to antibiotics (ATBs) before or during ICI treatment can affect the integrity of the GM and lead to intestinal dysbiosis, which has a profoundly negative impact on the treatment responses of various malignancies, including metastatic lung cancer, renal cell cancer melanoma and hepatocellular carcinoma.^10^, ^11^, ^12^, ^13^, ^14^, ^15^, ^16^, ^17^ However, most of these studies only included small samples or patients with various cancer types, rather than focusing specifically on SCLC. Thus, the association between the efficacy of ICIs and ATB exposure in ES‐SCLC remains unclear.

In this retrospective study, we compared the clinicopathological features and responses in a Chinese cohort of 214 ES‐SCLC patients who received ICIs with or without concomitant ATB treatment and analyzed the prognostic impact of ATB exposure on ICI outcomes.

## METHODS

### Patients

**FIGURE 1:**
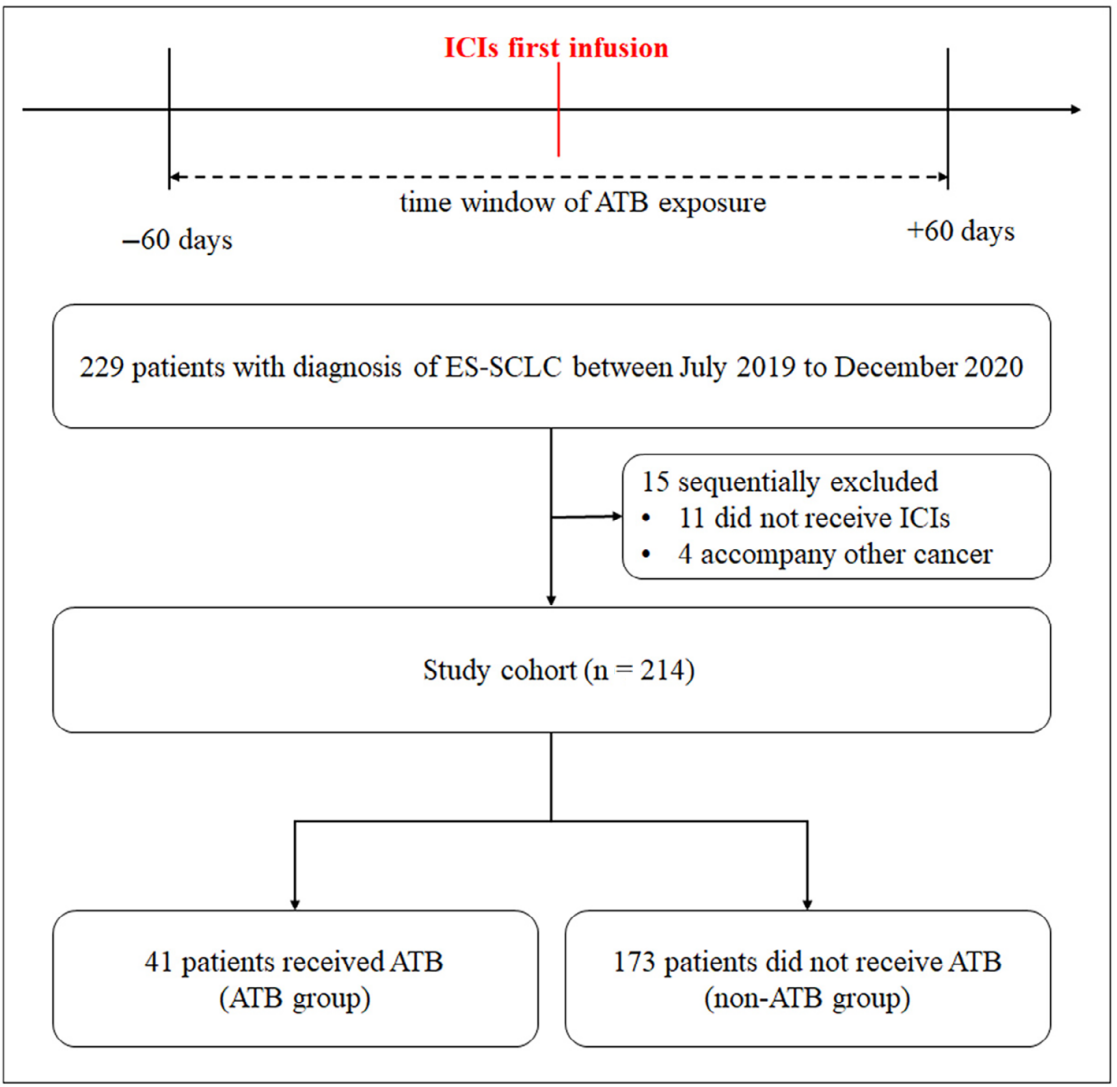
Patient selection flow diagram. ATB, antibiotic; ES‐SCLC, extensive‐stage small cell lung cancer; ICI, immune checkpoint inhibitor.

We reviewed the electronic medical records of all patients diagnosed with ES‐SCLC who started anti‐PD‐1/PD‐L1‐based therapies from July 2019 to December 2020 at Shandong Cancer Hospital and Institute, China. Figure 1 summarizes the patient selection process. Finally, 214 patients were evaluable for response assessment. All patients received ICIs as monotherapy, in combination with chemotherapy or angiogenesis inhibitors. The ICI information is shown in Table S1. Covariates for the statistical model were selected based on their prognostic values for ES‐SCLC, mainly including age, gender, Karnofsky performance status (KPS), nutritional risk screening 2002 (NRS2002), numerical rating scale (NRS), CAPRINI, body mass index (BMI), smoking history, drinking history, hypertension, diabetes mellitus, sites of metastases, and previous therapy. The therapeutic regimens, type (if any) of ATB used and the date of death or last follow‐up were recorded. The treatment was continued until disease progression, clinical deterioration, or unacceptable toxicity.

This study was approved by the ethics review board of the Shandong Cancer Hospital and Institute. The requirement for informed consent was waived given the retrospective nature of the study.

### Time window of ATB exposure

Previous studies examining the effect of ATB on immunotherapy outcomes used time windows varying from 2 weeks to 3 months, and the effect seems to depend on the time window of exposure, stronger effects being reported when the patients took ATB [−60 days; +60 days] around the first initiation of the ICI.^18^ Therefore, we hypothesized that a time window of ATB exposure within 2 months before or after the first infusion of ICIs for patients with ES‐SCLC would be associated with worse PFS and OS. The use of ATB was determined from information about concomitant medications recorded in the case report forms. All ATB classes were considered, including penicillin, cephalosporins, and quinolones (Table S2).

### Statistical analysis

Clinicopathological features were descriptively summarized by percentages. PFS was defined as the period from initiation of ICI treatment to the date of disease progression or death from any cause, whichever occurred first; OS was defined as the period from initiation of ICI therapy to the date of death from any cause. PFS and OS curves were drawn using the Kaplan–Meier method and were compared by the log‐rank test. Cox proportional hazards regression models were used to analyze the correlation of baseline clinical characteristics with the efficacy of ICIs. Possible factors associated with PFS and OS were first identified by a screening process via univariate Cox models. Variables with *p* < 0.05 were then tested in separate multivariate Cox models. Hazard ratios (HRs) estimated from the Cox analysis are reported with their 95% confidence intervals (CIs). All statistical analyses were conducted using SPSS version 27.0, GraphPad Prism version 9.5.1 and R version 4.2.1. All statistical tests were two‐sided, and *p* < 0.05 was the threshold for statistical significance.

## RESULTS

### Patient characteristics

A total of 214 patients were included in this study. We first dichotomized the patients according to ATB exposure: 41 (19.2%) received ATB within 2 months before or after the first initiation of ICI therapy (ATB group), and the remaining 173 patients did not receive ATB (non‐ATB group). The variables were well balanced across the ATB and non‐ATB groups, although there were greater proportions of brain metastasis (46.3% vs. 29.5%, *p* = 0.039) and thoracic radiotherapy (48.8% vs. 30.1%, *p* = 0.023) in the ATB group. KPS, NRS2002, NRS, CAPRINI and BMI at baseline between ATB and non‐ATB groups were observed no significantly difference indicating that there was no disparity in the health status at baseline. All patients received anti‐PD‐1/PD‐L1‐based immunotherapies. Of the included patients, 166 received ICIs plus chemotherapy, 17 received ICIs as monotherapy, 22 were treated with ICIs plus angiogenesis inhibitors (apatinib or anlotinib), and nine were treated with ICIs plus chemotherapy and angiogenesis inhibitors. The clinicopathological characteristics of all included patients are shown in Table 1.

**TABLE 1:** Patient characteristics.

Variables	Total (*n* = 214)	Non‐ATB (*n* = 173)	ATB (*n* = 41)	*p*‐value
Age (years), No (%)				
Median, range	60 (28–81)	60 (28–81)	59 (36–75)	
<65	144 (67.3)	118 (68.2)	26 (63.4)	0.556
≥65	70 (32.7)	55 (31.8)	15 (36.6)	
Gender, No (%)				
Male	163 (76.2)	130 (75.1)	33 (80.5)	0.470
Female	51 (23.8)	43 (24.9)	8 (19.5)	
KPS, No (%)				
<80	12 (5.6)	9 (5.2)	3 (7.3)	0.879
≥80	202 (94.4)	164 (94.8)	38 (92.7)	
NRS2002, No (%)				
Low nutritional risk (<3)	204 (95.3)	167 (96.5)	37 (90.2)	0.192
High nutritional risk (≥ 3)	10 (4.7)	6 (3.5)	4 (9.8)	
NRS, No (%)				
Low pain (1–3)	213 (99.5)	172 (99.4)	41 (100)	1.000
Moderate pain (4–6)	1 (0.5)	1 (0.6)	0 (0)	
CAPRINI, No (%)				
Low risk (0–2)	52 (24.3)	44 (25.4)	8 (19.5)	0.681
Moderate risk (3–4)	145 (67.8)	116 (67.1)	29 (70.7)	
High risk (≥5)	17 (7.9)	13 (7.5)	4 (9.8)	
BMI, No (%)				
Underweight (< 20)	14 (6.5)	14 (8.1)	0 (0)	0.160
Normal weight (20–25)	112 (52.3)	90 (52)	22 (53.7)	
Overweight (> 25)	88 (41.1)	69 (39.9)	19 (46.3)	
Smoking history, No (%)				
Never	92 (43)	77 (44.5)	15 (36.6)	0.357
Former/current	122 (57)	96 (55.5)	26 (63.4)	
Drinking history, No (%)				
Never	138 (64.5)	111 (64.2)	27 (65.9)	0.839
Former/current	76 (35.5)	62 (35.8)	14 (34.1)	
Hypertension, No (%)				
Yes	56 (26.2)	48 (27.7)	8 (19.5)	0.281
No	158 (73.8)	125 (72.3)	33 (80.5)	
Diabetes mellitus, No (%)				
Yes	32 (15)	24 (13.9)	8 (19.5)	0.363
No	182 (85)	149 (86.1)	33 (80.5)	
Sites of metastases, No (%)				
Brain	70 (32.7)	51 (29.5)	19 (46.3)	0.039
Liver	54 (25.2)	44 (25.4)	10 (24.4)	0.890
Adrenal	32 (15)	27 (15.6)	5 (12.2)	0.582
Bone	48 (22.4)	38 (22)	10 (24.4)	0.738
Lymph node	206 (96.3)	165 (95.4)	41 (100)	0.344
Previous therapy, No (%)				
Thoracic radio	72 (33.6)	52 (30.1)	20 (48.8)	0.023
Chemo	147 (68.7)	117 (67.6)	30 (73.2)	0.492
Antiangiogenesis	36 (16.8)	29 (16.8)	7 (17.1)	0.962
Therapeutic regimen, No (%)				
ICIs mono	19 (8.9)	14 (8.1)	5 (12.2)	0.867
ICIs + chemo	164 (76.6)	134 (77.5)	30 (73.2)	
ICIs + antiangiogenesis	22 (10.3)	18 (10.4)	4 (9.8)	
ICIs + chemo + antiangiogenesis	9 (4.2)	7 (4)	2 (4.9)	

Abbreviations: ATB, antibiotic; BMI, body mass index; chemo, chemotherapy; KPS, Karnofsky performance status; mono, monotherapy; No, number; NRS, numerical rating scale; NRS2002, nutritional risk screening 2002; radio, radiotherapy.

### ATB treatment characteristics

A total of 41 patients (19.2%) received at least one dose of ATB. In six of them (14.62%), more than one ATB was administered. The most frequently prescribed ATB was penicillin (20 cases, 41.7%), followed by quinolones (11 cases, 22.9%), cephalosporins (10 cases, 22.8%), carbapenems (3 cases, 6.3%), glycopeptides (2 cases, 4.2%), aminoglycosides (1 case, 2.1%), and macrolides (1 case, 2.1%) (Table S2). ATB was administered intravenously route in 39 cases (95.12%) and orally route in two cases (4.88%). The most common indication for ATB treatment was pneumonitis, which occurred in 31 (75.6%) of the patients in the ATB group (Table S3).

### Correlation between ATB exposure and clinical outcomes

**FIGURE 2:**
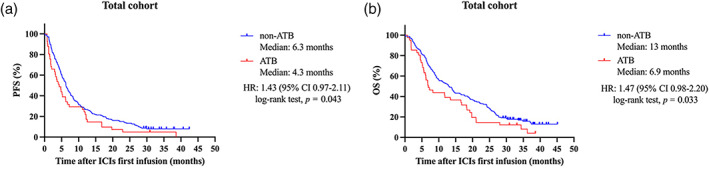
(a) Progression‐free survival (PFS) and (b) overall survival (OS) among all patients, stratified by ATB exposure. CI, confidence interval; HR, hazard ratio; ICIs, immune checkpoint inhibitors; OS, overall survival; PFS, progression‐free survival.

**FIGURE 3:**
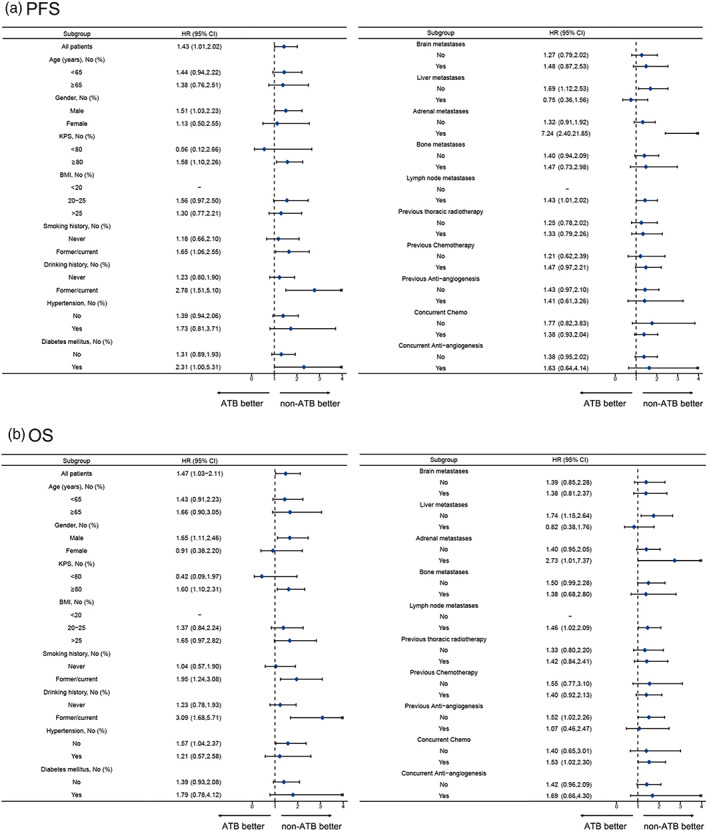
Forest plot of subgroup analysis by baseline characteristics for PFS (a) and OS (b) among all patients. ATB, antibiotic; CI, confidence interval; HR, hazard ratio; OS, overall survival; PFS, progression‐free survival.

We first assessed the impact of ATB exposure on clinical outcomes in the total cohort. In the analysis of all 214 patients, the ATB group showed a shorter mPFS: 4.3 months versus 6.3 months in the non‐ATB group (HR 1.43, 95% CI: 0.97–2.11; log‐rank test, *p* = 0.043, Figure 2a). The ATB group also had a shorter mOS (6.9 vs. 13 months; HR 1.47, 95% CI: 0.98–2.20; log‐rank test, *p* = 0.033, Figure 2b). We further investigated the impact of ATB exposure on survival according to immunotherapeutic regimen (Figure S1). We also conducted subgroup analyses based on various clinicopathological factors. The results were consistent with those of the total cohort analyses, with OS and PFS being superior in the non‐ATB group in most of the analyses (Figure 3a,b).

### Multivariate analysis

We performed univariate and multivariate Cox regression analyses to explore the potential clinical and pathological parameters that may be associated with PFS or OS (Tables 2 and 3). In univariate analysis, we found that patients with concomitant ATB treatment had significantly shorter PFS (HR = 1.43, 95% CI: 1.01–2.02, *p* = 0.044) and OS (HR = 1.47, 95% CI: 1.03–2.11, *p* = 0.034) than patients without ATB treatment. Patients with diabetes mellitus (PFS: HR = 1.59, 95% CI: 1.09–2.33, *p* = 0.016; OS: HR = 1.63, 95% CI: 1.10–2.41, *p* = 0.016) or metastases (PFS: HR = 1.29, 95% CI: 1.12–1.48, *p* < 0.001; OS: HR = 1.33, 95% CI: 1.15–1.54, *p* < 0.001) also had worse PFS and OS. Female patients had better OS (HR = 0.67, 95% CI: 0.47–0.96, *p* = 0.027). These significant factors, together with the variables with *p* < 0.05 identified in univariable analysis, were included in multivariate analysis for both PFS and OS. Importantly, concomitant ATB treatment remained an independent prognostic factor for PFS (HR = 1.47, 95% CI: 1.03–2.09, *p* = 0.035) and OS (HR = 1.46, 95% CI: 1.01–2.11, *p* = 0.043) after adjusting for other confounding factors. Diabetes mellitus (PFS: HR = 1.72, 95% CI: 1.16–2.54, *p* = 0.006; OS: HR = 1.66, 95% CI: 1.11–2.49, *p* = 0.014), metastases (PFS: HR = 1.27, 95% CI: 1.10–1.47, *p* = 0.001; OS: HR = 1.28, 95% CI: 1.10–1.50, *p* = 0.002) and previous therapy (PFS: HR = 1.88, 95% CI: 1.37–2.58, *p* < 0.001; OS: HR = 1.51, 95% CI: 1.09–2.11, *p* = 0.015) were significantly associated with worse PFS and OS.

**TABLE 2:** Univariate and multivariate cox regression analyses of clinical parameters on PFS.

Prognostic parameters	Univariate analysis		Multivariate analysis	
	HR (95% CI)	*p*‐value	HR (95% CI)	*p*‐value
Age (years)				
<65	1.00			
≥65	0.88 (0.65–1.18)	0.388		
Gender				
Male	1.00			
Female	0.73 (0.52–1.02)	0.064		
KPS				
<80	1.00			
≥80	0.89 (0.49–1.64)	0.716		
BMI				
<20	1.00	0.924		
20–25	0.99 (0.56–1.76)	0.972		
>25	0.94 (0.52–1.68)	0.825		
NRS2002				
Low nutritional risk (<3)	1.00			
High nutritional risk (≥ 3)	1.26 (0.64–2.45)	0.506		
NRS				
Low pain (1–3)	1.00			
Moderate pain (4–6)	0.05 (0–12.92)	0.281		
CAPRINI				
Low risk (0–2)	1.00	0.468		
Moderate risk (3–4)	0.82 (0.59–1.13)	0.221		
High risk (≥5)	0.90 (0.52–1.56)	0.704		
Smoking history				
Never	1.00		1.00	
Former/current	1.51 (1.13–2.02)	0.005	1.41 (0.98–2.04)	0.068
Drinking history				
Never	1.00		1.00	
Former/current	1.36 (1.02–1.83)	0.037	1.28 (0.88–1.85)	0.202
Hypertension				
No	1.00			
Yes	1.07 (0.78–1.46)	0.691		
Diabetes mellitus				
No	1.00		1.00	
Yes	1.59 (1.09–2.33)	0.016	1.72 (1.16–2.54)	0.006
ATB treatment				
No	1.00		1.00	
Yes	1.43 (1.01–2.02)	0.044	1.47 (1.03–2.09)	0.035
Number of metastases sites (ordinal data, from 0 to 4)	1.29 (1.12–1.48)	< 0.001	1.27 (1.10–1.47)	0.001
Previous therapy				
No	1.00		1.00	
Yes	1.62 (1.19–2.20)	0.002	1.88 (1.37–2.58)	<0.001
Therapeutic regimen				
ICIs mono		0.741		
ICIs + chemo	1.13 (0.68–1.87)	0.632		
ICIs + antiangiogenesis	1.03 (0.54–1.97)	0.932		
ICIs + chemo +antiangiogenesis	0.79 (0.34–1.82)	0.357		
Concurrent chemo				
No				
Yes	1.09 (0.76–1.56)	0.639		
Concurrent antiangiogenesis				
No				
Yes	0.85 (0.57–1.26)	0.417		

Abbreviations: ATB, antibiotic; BMI, body mass index; chemo, chemotherapy; CI, confidence interval; HR, hazard ratio; ICIs, immune checkpoint inhibitors; KPS, Karnofsky performance status; mono, monotherapy; NRS, numerical rating scale; NRS2002, nutritional risk screening 2002; PFS, progression‐free survival; radio, radiotherapy.

**TABLE 3:** Univariate and multivariate cox regression analyses of clinical parameters on OS.

Prognostic parameters	Univariate analysis		Multivariate analysis	
	HR (95% CI)	*p*‐value	HR (95% CI)	*p*‐value
Age (years)				
<65	1.00			
≥65	1.13 (0.83–1.53)	0.447		
Gender				
Male	1.00		1.00	
Female	0.67 (0.47–0.96)	0.027	0.90 (0.58–1.41)	0.645
KPS				
<80	1.00			
≥80	0.67 (0.37–1.24)	0.204		
BMI				
<20	1.00			
20–25	0.78 (0.44–1.39)	0.396		
>25	0.78 (0.43–1.41)	0.410		
NRS2002				
Low nutritional risk (<3)	1.00			
High nutritional risk (≥ 3)	1.53 (0.78–2.99)	0.216		
NRS				
Low pain (1–3)	1.00			
Moderate pain (4–6)	0.05 (0.00–19.29)	0.321		
CAPRINI				
Low risk (0–2)	1.00	0.422		
Moderate risk (3–4)	0.82 (0.59–1.15)	0.244		
High risk (≥5)	0.73 (0.40–1.33)	0.304		
Smoking history				
Never	1.00		1.00	
Former/current	1.49 (1.11–2.01)	0.008	1.19 (0.78–1.80)	0.424
Drinking history				
Never	1.00		1.00	
Former/current	1.57 (1.17–2.12)	0.003	1.50 (1.03–2.20)	0.037
Hypertension				
No	1.00			
Yes	1.28 (0.93–1.76)	0.133		
Diabetes mellitus				
No	1.00		1.00	
Yes	1.63 (1.10–2.41)	0.016	1.66 (1.11–2.49)	0.014
ATB treatment				
No	1.00		1.00	
Yes	1.47 (1.03–2.11)	0.034	1.46 (1.01–2.11)	0.043
Number of metastases sites (ordinal data, from 0 to 4)	1.33 (1.15–1.54)	< 0.001	1.28 (1.10–1.50)	0.002
Previous therapy				
No	1.00		1.00	
Yes	1.38 (0.10–1.90)	0.05	1.51 (1.09–2.11)	0.015
Therapeutic regimen				
ICIs mono	1.00			
ICIs + chemo	1.51 (0.87–2.58)	0.129		
ICIs + antiangiogenesis	1.46 (0.74–2.90)	0.278		
ICIs + chemo+ antiangiogenesis	0.84 (0.33–2.17)	0.719		
Concurrent chemo				
No	1.00			
Yes	1.21 (0.83–1.21)	0.323		
Concurrent antiangiogenesis				
No	1.00			
Yes	0.86 (0.56–1.32)	0.483		

Abbreviations: ATB, antibiotic; BMI, body mass index; chemo, chemotherapy; CI, confidence interval; HR, hazard ratio; ICIs, immune checkpoint inhibitors; KPS, Karnofsky performance status; mono, monotherapy; NRS, numerical rating scale; NRS2002, nutritional risk screening 2002; OS, overall survival; radio, radiotherapy.

## DISCUSSION

To our knowledge, this is the first study to demonstrate the potential harmful effects of ATB exposure in Chinese ES‐SCLC patients receiving anti‐PD1/PD‐L1‐based immunotherapy. Our results confirm that ATB exposure within 2 months before or after the first infusion of ICIs is significantly associated with worse PFS and OS in ES‐SCLC patients.

With the increasing use of ICIs for various malignancies, much effort has been made to identify reliable biomarkers that may predict the efficacy of ICIs, and ATB exposure has recently emerged as one of them. Our findings are consistent with studies reporting adverse effects of ATB in patients receiving ICIs for NSCLC,^10^, ^11^, ^12^, ^14^, ^15^ melanoma,^13^, ^15^ renal cell carcinoma,^11^ hepatocellular carcinoma,^16^, ^17^ and other tumor types.

The dysbiosis of the GM has been associated with various human health conditions and diseases, such as extraintestinal autoimmune diseases,^19^ Alzheimer's disease,^20^ Parkinson's disease,^21^ and asthma.^22^ Accumulating evidence suggests that a loss of diversity or a shift in the composition of the GM can attenuate the therapeutic efficacy of ICIs, indicating that the diversity of the GM may play a prominent role in modulating the tumor response to ICI therapy.^7^, ^9^, ^23^, ^24^, ^25^ Gopalakrishnan V et al.^7^ reported that patients with a favorable diversity of GM have enhanced systemic and antitumor immune responses mediated by increased antigen presentation and improved effector T cell function in the periphery and the tumor microenvironment in melanoma patients in the Western population. Routy et al.^9^ found that primary resistance to ICIs can be attributed to abnormal GM composition, and fecal microbiota transplantation (FMT) from cancer patients who responded to ICIs into germ‐free or antibiotic‐treated mice ameliorated the antitumor effects of PD‐1 blockade, whereas FMT from nonresponding patients failed to do so, potentially highlighting the central role of the GM in driving adaptive resistance to ICIs.^26^ Using the gold‐standard 16S ribosomal RNA sequence, previous studies have found that the enrichment of certain stool bacterial species, including Ruminococcus, Akkermansia, and Bifidobacteria, is associated with a higher likelihood of response to ICIs.^8^, ^17^, ^27^ ATB exposure has been addressed as a detrimental predictor of ICI efficacy, being associated with the highest disruption of and potential to induce long‐lasting changes to the GM by causing such profound changes as a decrease in bacterial diversity,^28^ changes in the abundances of certain bacteria^29^ and impairment of the effectiveness of the cytotoxic T cell response against cancer.^30^ Owing to the geography, ethnicity and subsistence‐specific variations in human GM composition and diversity,^31^ the impact of ATB exposure on ICI efficacy still needs to be confirmed in different patient cohorts.

We also investigated the impact of ATB exposure under different immunotherapeutic regimens, and our data demonstrated a negative effect of ATB exposure on PFS (HR 1.37, 95% CI: 0.87–2.15; Gehan‐Breslow Wilcoxon test, *p* = 0.045, Figure S1A) and OS (HR 1.54, 95% CI: 0.95–2.48; Gehan‐Breslow Wilcoxon test, *p* = 0.0216, Figure S1B) in patients receiving ICIs plus chemotherapy, as expected. However, no significant difference in PFS or OS was observed in patients who received ICI monotherapy, ICI plus angiogenesis inhibitors, or ICI plus chemotherapy and angiogenesis inhibitors (see Figure S1C–H). Notably, relatively few and heterozygous patients were included in the subgroup analysis. Among the 19, 22, and nine patients in the ICI monotherapy, ICI plus angiogenesis inhibitors, ICI plus chemotherapy and angiogenesis inhibitors cohorts, respectively, only five, four, and two patients were identified in the ATB group. We speculate that the potential imbalance of patient selection and the small sample size might have confounded the survival analysis. Due to the limited data on the prognostic impact of ATB on the combination of ICIs and chemotherapy or angiogenesis inhibitors in lung cancer, larger studies are undoubtedly needed to better understand this phenomenon. Proton pump inhibitors (PPIs), another medication class that can alter the composition of the GM, have also been evaluated for their influence on ICI efficacy.^10^, ^32^ PPIs are also widely used in patients with cancer to prevent oversecretion of gastric acid and indigestion, which are often induced by chemotherapy. In our cohort, most patients received ICIs plus chemotherapy with concomitant PPIs, so we did not include PPIs in our analyses.

The limitations in the present study must be acknowledged. First, this was a retrospective study performed at a single cancer center, making selection bias inevitable. Several discrepancies in baseline characteristics were observed, including a higher rate of brain metastasis and more patients treated with thoracic radiotherapy in the ATB group, which might have confounded our analysis. To compensate for these discrepancies, we evaluated the impact of ATB treatment across individual subgroups and performed multivariable analysis to adjust for multiple prognostic factors. Second, as the patients included in this study were only representative of a single nation, it is difficult to extrapolate the findings to other ethnic populations. In addition, the population was slightly heterogeneous regarding the treatment regimen received, which was attributed to some reasons such as intolerance to chemotherapy, economic hardship, and refusal of treatment by patients. Moreover, we failed to investigate the impact of ATB based on biomarkers, such as PD‐L1 expression, TMB and TILs, because the detection of these biomarkers was not mandatory for ES‐SCLC patients before they received ICIs, which might also skew the clinical responses and survival benefits. In addition, the timing and duration of ATB treatment were not clear. Last, based on growing evidence in the literature and in the clinic, we assumed that antimicrobials may cause an imbalance in the GM and consequently diminish the effectiveness of ICIs. However, this study cannot discuss causality between ATB exposure and impaired clinical outcomes of ES‐SCLC patients treated with ICIs, nor can it elucidate the underlying biological mechanisms involved. It can only show an association between ATB exposure and reduced ICI efficacy. As it is not ethically feasible to conduct interventional, randomized, controlled trials in which ATB would be administered to cancer patients treated with ICIs to demonstrate their deleterious impact when compared to their absence, prospective observational studies and interventional trials of microbiome modifiers are urgently needed to uncover the role of the microbiome and improve patient outcomes by carefully recording ATB dosing and important confounders and collecting samples before and after antimicrobial treatment for biomarker discovery and mechanistic exploration. Such studies will reduce the existing publication bias by allowing analyses on more homogeneous populations, especially in terms of treatments received, which is not possible at this stage given the current state of the field. Until then, ATB prescriptions should be cautiously considered in cancer patients receiving ICIs.

In conclusion, ATB exposure is associated with worse survival in ES‐SCLC patients receiving ICI therapy. Given the known overutilization of ATBs in the world today, ATB should be prescribed cautiously in ES‐SCLC patients receiving ICI therapy.

## AUTHOR CONTRIBUTIONS

Jiaqi Zhong: conceptualization, data curation, methodology, software, formal analysis, writing ‐ original draft; Dali Xiong: data curation, writing ‐ original draft; Yu Liu: supervision; Shuanghu Yuan: Writing ‐ review and editing.

## FUNDING INFORMATION

This study was supported in part by the National Natural Science Foundation of China (NSFC82073345), Natural Science Innovation and Development Joint Foundation of Shandong Province (ZR202209010002), Jinan Clinical Medicine Science and Technology Innovation Plan (202019060) and the Taishan Scholars Program to Shuanghu Yuan.

## CONFLICT OF INTEREST STATEMENT

The authors declare no potential conflicts of interest.

## Supporting information


**Appendix A.** Supplementary data.

## Data Availability

The datasets used and/or analyzed during the current study are available from the corresponding author on reasonable request.
