# Supplementary material for: Association of antibiotic exposure with survival in patients with extensive‐stage small cell lung cancer receiving immune checkpoint inhibitor therapy
Source: Thorac Cancer. 2023 Nov 27;15(2):152–62. doi: 10.1111/1759-7714.15172 (PMC10788467; doi:10.1111/1759-7714.15172)
Supplement: Supplementary file 1 — Appendix A. Supplementary data. [file TCA-15-152-s001.docx]

**Appendix A. Supplementary data**

- TABLES

**Supplementary Table 1. Use of ICIs administered in this study.**

**Supplementary Table 2. Use of ATB within window in the randomized population.**

**Supplementary Table 3. Indications and routes of ATB use.**

**Supplementary Table 4. Characteristics on patients with ICIs in combination with angiogenesis inhibitors (N=31).**

- FIGURES

**Supplementary Fig. 1. The correlations of concomitant ATB exposure and clinical outcomes during different immunotherapeutic regimens.**

**Supplementary Fig. 2. Clinical outcomes of patients receiving ICIs in combination with angiogenesis inhibitors.**

**Supplementary Table 1. Use of ICIs administered in this study.**

| **ICIs** | | **number (%)** |
| --- | --- | --- |
| **PD-L1** | Atezolizumab | 44 (20.6) |
|  | Durvalumab | 45 (21) |
| **PD-1** | Camrelizumab | 26 (12.1) |
|  | Nivolumab | 12 (5.6) |
|  | Pembrolizumab | 4 (1.9) |
|  | Penpulimab | 2 (0.9) |
|  | Toripalimab | 20 (9.3) |
|  | Tislelizumab | 12 (5.6) |
|  | Sintilimab | 49 (22.9) |
| **Total number (%)** |  | 214 (100) |

**Supplementary Table 2. Use of ATB within window in the randomized population.**

| **Antibiotics** | **number (%)** |
| --- | --- |
| Penicillins | 20 (41.7) |
| Quinolone | 11 (22.9) |
| Cephalosporin | 10 (22.8) |
| Carbapenem | 3 (6.3) |
| Glycopeptide | 2 (4.2) |
| Aminoglycoside | 1 (2.1) |
| Macrolide | 1 (2.1) |
| *A patient can be counted more than once. | |

**Supplementary Table 3. Indications and routes of ATB use.**

| **Indications** | **Number (%)** |
| --- | --- |
| Pneumonia | 31 (75.6) |
| Fever of unknown cause or empirical use | 4 (9.8) |
| surgery | 2 (4.9) |
| Intestinal infection | 1 (2.4) |
| Gingivitis | 1 (2.4) |
| maxillary sinusitis | 1 (2.4) |
| Biliary tract infection | 1 (2.4) |
| **Routes** | **Number (%)** |
| Intra-venous route | 39 (95.1) |
| Oral route | 2 (4.9) |
| **Supplementary Table 4. Characteristics on patients with ICIs in combination with angiogenesis inhibitors (N=31).** | |
| **Variables** |  |
| **Age (years), No (%)** |  |
| Median, Range | 56, 38-77 |
| < 65 | 21 (67.7) |
| ≥ 65 | 10 (32.3) |
| **Gender, No (%)** |  |
| Male | 22 (71) |
| Female | 9 (29) |
| **KPS, No (%)** |  |
| < 80 | 2 (6.5) |
| ≥80 | 29 (93.5) |
| **NRS2002, No (%)** |  |
| Low nutritional risk (< 3) | 30 (96.8) |
| High nutritional risk (≥ 3) | 1 (3.2) |
| **NRS, No (%)** |  |
| Low pain (1-3) | 30 (96.8) |
| Moderate pain (4-6) | 1 (3.2) |
| **CAPRINI, No (%)** |  |
| Low risk (0-2) | 4 (12.9) |
| Moderate risk (3-4) | 25 (80.6) |
| High risk (≥ 5) | 2 (6.5) |
| **BMI, No (%)** |  |
| Underweight (< 20) | 1 (3.2) |
| Normal weight (20-25) | 12 (38.7) |
| Overweight (> 25) | 18 (58.1) |
| **Smoking history, No (%)** |  |
| Never | 16 (51.6) |
| Former/current | 15 (48.4) |
| **Drinking history, No (%)** |  |
| Never | 23 (74.2) |
| Former/current | 8 (25.8) |
| **Hypertension, No (%)** |  |
| Yes | 22 (71) |
| No | 9 (29) |
| **Diabetes mellitus, No (%)** |  |
| Yes | 25 (80.6) |
| No | 6 (19.4) |
| **Sites of metastases, No (%)** |  |
| Brain | 13 (41.9) |
| Liver | 8 (25.8) |
| Adrenal | 3 (9.7) |
| Bone | 5 (16.1) |
| Lymph node | 31 (100) |
| **Previous therapy, No (%)** |  |
| Thoracic radio | 15 (48.4) |
| Chemo | 30 (96.8) |
| Anti-angiogenesis | 9 (29) |
| **Therapeutic regimen, No (%)** |  |
| ICIs+ Anti-angiogenesis | 22 (71) |
| ICIs+ Chemo+ Anti-angiogenesis | 9 (29) |
| Abbreviations: OS, overall survival; HR, hazard ratio; CI, confidence interval; KPS, karnofsky performance status; NRS2002, nutritional risk screening 2002; NRS, numerical rating scale; BMI, body mass index; ATB, antibiotic; ICIs, immune checkpoint inhibitors; radio, radiotherapy; Chemo, chemotherapy; mono, monotherapy. | |

**Supplementary Fig. 1. The correlations of concomitant ATB exposure and clinical outcomes during different immunotherapeutic regimens.**


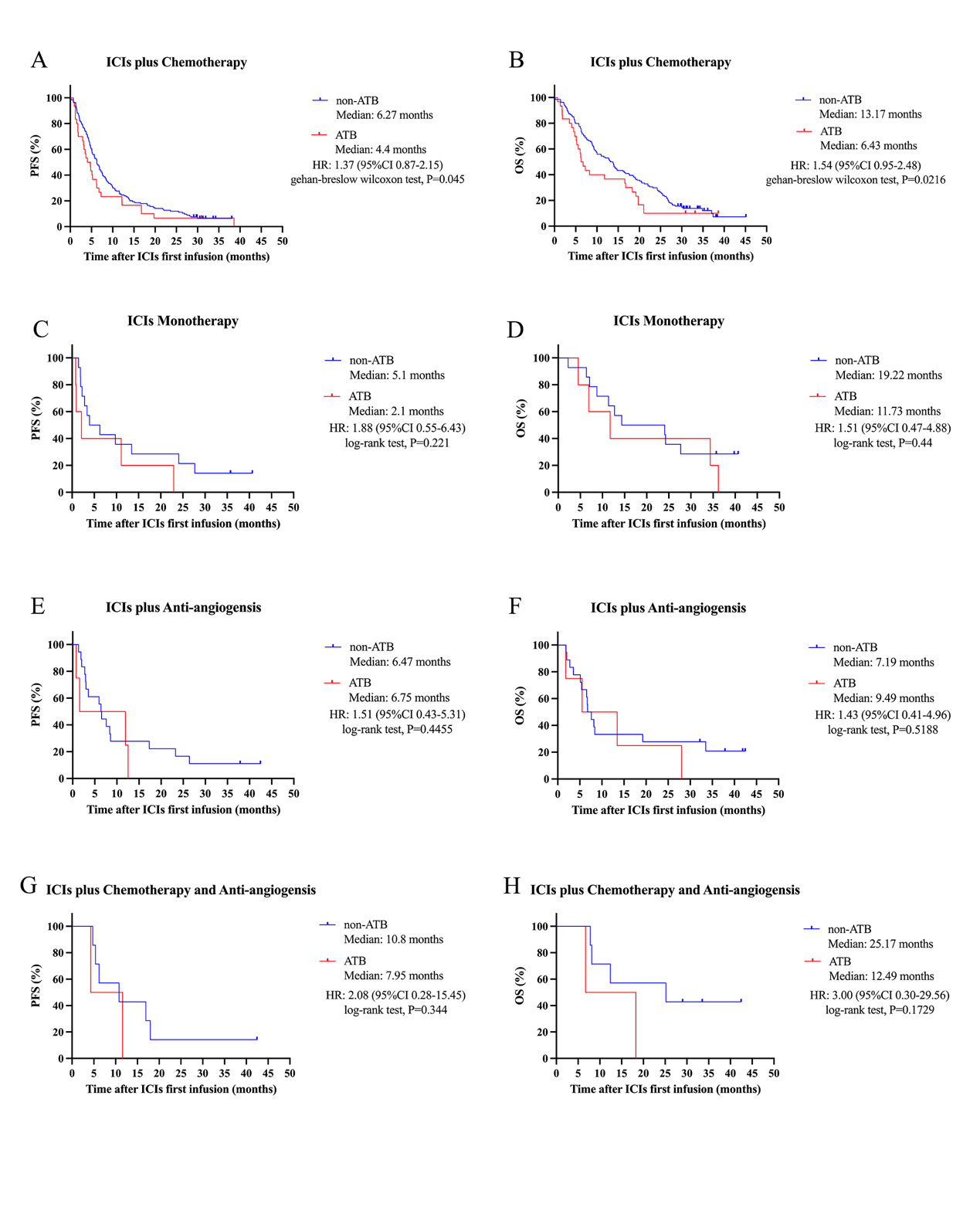
 **Supplementary Fig. 2. Clinical outcomes of patients receiving ICIs in combination with angiogenesis inhibitors.**

**
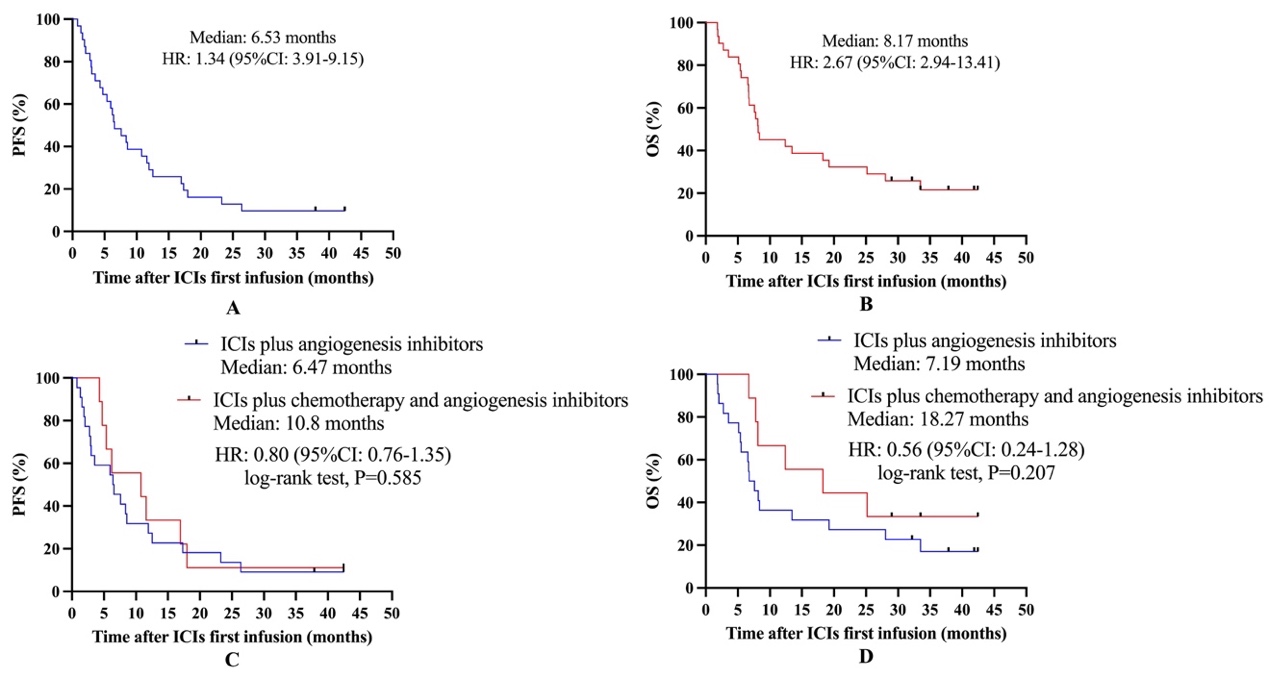
**
